# A membrane‐bound esterase PA2949 from *Pseudomonas aeruginosa* is expressed and purified from *Escherichia coli*

**DOI:** 10.1002/2211-5463.12061

**Published:** 2016-04-19

**Authors:** Filip Kovacic, Florian Bleffert, Muttalip Caliskan, Susanne Wilhelm, Joachim Granzin, Renu Batra‐Safferling, Karl‐Erich Jaeger

**Affiliations:** ^1^ Institute of Molecular Enzyme TechnologyHeinrich‐Heine Universität Düsseldorf, Forschungszentrum JülichGermany; ^2^ Institute of Complex Systems, ICS‐6: Structural BiochemistryForschungszentrum JülichGermany; ^3^ Institute of Bio‐ and Geosciences IBG‐1: BiotechnologyForschungszentrum Jülich GmbHGermany

**Keywords:** (*D*)‐*β*‐acetylthioisobutyric acid, *Escherichia coli*, esterase, inhibition, membrane protein, *Pseudomonas aeruginosa*

## Abstract

*Pseudomonas aeruginosa* strain 1001 produces an esterase (EstA) that can hydrolyse the racemic methyl ester of β‐acetylthioisobutyrate to produce the (*D*)‐enantiomer, which serves as a precursor of captopril, a drug used for treatment of hypertension. We show here that PA2949 from *P. aeruginosa* PA01, a homologue of EstA, can efficiently be expressed in an enzymatically active form in *E. coli*. The enzyme is membrane‐associated as demonstrated by cell fractionation studies. PA2949 was purified to homogeneity after solubilisation with the nonionic detergent, Triton X‐100, and was shown to possess a conserved esterase catalytic triad consisting of Ser137–His258–Asp286. Our results should allow the development of an expression and purification strategy to produce this biotechnologically relevant esterase in a pure form with a high yield.

**Abbreviations**
DAT: (*D*)‐*β*‐acetylthioisobutyric acid
EDTA: ethylenediaminetetraacetic acid
IMAC: immobilised metal affinity chromatography
MUB: 4‐methylumbelliferyl butyrate
PMSF: phenylmethylsulfonyl fluoride
*p*NPC_6_: *p*‐nitrophenyl hexanoate
*p*NPP: *p*‐nitrophenyl palmitate
SDS/PAGE: sodium dodecyl sulphate polyacrylamide gel electrophoresis
THL: tetrahydrolipstatine

Carboxyl esterases (EC 3.1.1.1) and lipases (EC 3.1.1.3), usually referred to as lipolytic enzymes, hydrolyse ester bonds of a wide range of lipidic substrates 1. They exert various cellular functions in animals, plants and microorganisms 2, 3, 4. The present classification scheme consists of 15 bacterial lipase families, which contain several hundreds of protein sequences and three dimensional structures 5, 6. Despite relatively low amino acid sequence similarity (typically below 20%) lipolytic enzymes have a common α/β‐hydrolase fold, characterised by a central hydrophobic sheet composed of mostly eight β‐strands, which are connected by α‐helices 7. These enzymes are characterised by a typical GXSXG‐consensus motif containing the catalytic serine, which forms the catalytic triad with histidine and aspartate residues 8. Glycine residues of the GXSXG‐motif account for the localisation of the catalytic serine on top of a sharp turn preceded by an α‐helix followed by a β‐strand. This structural motif, named nucleophilic elbow, is essential for substrate hydrolysis and represents one of the best conserved structural motifs among α/β‐hydrolases. The catalytic triad and the residues forming the oxyanion hole, which stabilises the tetrahedral intermediates formed during the hydrolysis, are structurally strongly conserved in all α/β‐hydrolases 9.

Although many structural features of α/β‐hydrolases are similar, these enzymes show a wide range of functionalities 10. In recent years, the number of biochemical and structural data about lipolytic enzymes are rapidly increased, however, the understanding of structure‐function relationship is still limited 11. Nowadays, lipolytic enzymes are broadly used as industrial biocatalysts, since many of them are fairly stable in harsh conditions that are often needed for industrial bioprocess 12, 13, for example, high temperatures, presence of organic solvents or high ionic strength buffers. Moreover, their enantio‐, stereo‐ and regioselectivity allow controlled organic synthesis reactions using complex unnatural substrates, as, for example, precursors for the biopharmaceuticals paclitaxel 14, naproxen 15 and captopril 16. The latter is the first drug used for the treatment of hypertension, congestive heart failure and diabetic nephropathy, which acts by inhibiting angiotensin‐converting enzyme 16. Presently, captopril is still prescribed and distributed under the trade name Capoten^®^. Its chemical synthesis requires the optically pure key intermediate, (*D*)‐*β*‐acetylthioisobutyric acid (DAT) as the inhibiting potency of the (*D*)‐enantiomer is 100‐fold higher than of the (*L*)‐enantiomer 16, 17. To overcome the laborious and expensive chemical synthesis of optically active DAT, Sakimae *et al*. 18 screened for microorganisms producing esterases, which can stereoselectively hydrolyse the racemic methyl ester of *β*‐acetylthioisobutyrate 18. The functional screening revealed several strains capable of DAT synthesis with *Pseudomonas* strains producing DAT of highest optical purity 18.

The esterase EstA from *P. aeruginosa* 1001 was cloned, overexpressed and shown to produce DAT 19, 20. However, expression of EstA in *E. coli* resulted in low enzymatic activity presumably caused by formation of protein aggregates, which could not be completely abolished by fusion with maltose‐binding protein 19, 20. Here, we report cloning, expression and purification of the protein encoded by open reading frame *pa2949* from *P. aeruginosa* PA01, which is homologous to EstA from *P. aeruginosa* 1001. We have developed a system for efficient expression of highly active PA2949 in common laboratory strain *E. coli* BL21(DE3). Biochemical analysis showed esterase but no lipase activity of PA2949, and enzyme activity could be inhibited by serine‐hydrolase inhibitors. Furthermore, we showed that PA2949 is localised in the membrane of *E. coli* prompting us to develop a detergent‐based purification method, which yielded mg amounts of enzymatically active protein. Our data demonstrates that PA2949 can be functionally expressed, easily purified and adequately stabilised thus making it available for a range of different biotechnological applications.

## Materials and methods

### Bioinformatic analysis

Amino acid sequences were analysed and aligned using the blast search and alignment tool of the Universal Protein Knowledge Base (www.uniprot.org) 21. Signal peptide cleavage sites were predicted by two different methods, namely the Hidden Markov Model (Signal P‐HMM) 22 and neural network (Signal P‐NN) 22. Signal peptides were distinguished from nonsignal peptides by a threshold *D*‐score of SignalP‐NN higher than 0.5 and by a threshold *C*‐score of SignalP‐HMM higher than 0.95. The transmembrane helix was predicted using the Toppred 23 online tool with a score higher than 0.8.

### Cloning, site‐directed mutagenesis, expression and purification of PA2949

Restriction endonucleases, *Pfu* DNA polymerase and bacteriophage T4 DNA ligase (Thermo Scientific, Darmstadt, Germany) reactions were carried out as recommended by the manufacturers. DNA fragments were analysed on 1% (w/v) agarose gels. Plasmid DNA was purified using the InnuPREP DOUBLE pure kit (Analytik Jena, Jena, Germany) or, for genomic DNA from *P. aeruginosa* PA01 using the DNeasy tissue kit (Qiagen, Hilden, Germany). Used strains and plasmids are listed in Table 1 24, 25, 26, 27.

**Table 1:** Strains and plasmids used in this study

Strains	Genotype	Source
*E. coli* DH5α	*sup*E44 Δ(*lac*ZYA‐*arg*F)U196 (Φ80Δ*lac*ZM15) *hsd*R17 *rec*A1 *end*A1 *gyr*A96 *thi*‐1 *rel*A1	24
*E. coli* BL21(DE3)	F^−^ *omp*T *hsd*SB(r_B_^−^m_B_^−^) *gal dcm* (λIts857 *ind*I *sam*7 *nin*5 *lac*UV5‐T7gene1)	25
*P. aeruginosa* PA01	Wild‐type, originating from Dieter Haas laboratory (Lausanne, CH)	26

Plasmids	Description	Source
pET22b+	*Col*E1 PT7_Ф10_ *pel*B Ap^r^ C‐His_6_‐‘Tag’ *lac*I^q^	Novagen
pBBR1mcs3	Cm^r^ mob *lac*Zα P*lac* PT7 Tc^r^	27
pET22b‐*pa2949*	*pa2949H6* gene inserted in *Nde*I/*Sac*I of pET22b(+)	This study
pET22b‐*pa2949*_S137A	*pa2949* gene with substitutions of Ser137 with Ala inserted in *Nde*I/*Sac*I sites of pET22b(+)	This study
pBBR1mcs3‐*pa2949*	*Xba*I/*Sac*I fragment of pET22b‐*pa2949* inserted in pBBR1mcs‐3	This study

The *pa2949* gene was amplified by standard PCR using *Pfu* DNA polymerase, the genomic DNA of *P. aeruginosa* PA01 as a template and oligonucleotide pair 5′‐AAACATATGAAACGATTCCTC‐3′/5′‐TCAGAGCTC **CACCACCACCACCACCAC**GCGACCGGCCAC‐3′ encoding *Nde*I and *Sac*I restriction sites (underlined) and a C‐terminal His_6_‐tag (bold). Primers were synthesised by MWG Biotech. The *pa2949* gene was cloned into *Nde*I and *Sac*I restriction sites of pET22b yielding expression plasmid pET‐*pa2949* (Table 1) allowing for bacteriophage T7‐RNA polymerase‐dependent expression from the T7 promoter. The mutation of Ser137Ala in PA2949 was performed by the Quik‐change PCR method using *Pfu* DNA polymerase (Invitrogen, Darmstadt, Germany), pET‐*pa2949* plasmid and the complementary mutagenic oligonucleotide pair 5′‐TGGCCGGCAACG ^T^ C ^C^ C ^C^ATGGGCGGG‐3′/5′‐CCCGCCCATG ^G^ G ^G^ C ^A^GTTGCCGGCCA‐3′ (mutated codons are underlined and nucleotides of the wild‐type gene are indicated in the subscript). Correctness of plasmids pET‐*pa2949* and pET‐*pa2949*_S137A was confirmed by DNA sequencing (MWG Biotech, Ebersberg, Germany).

For the expression of PA2949 and PA2949 S137A, *E. coli* BL21(DE3) cells transformed respectively with pET‐*pa2949* and pET‐*pa2949*_S137A were grown overnight at 37 °C in a Luria–Bertani (LB) medium supplemented with ampicillin (100 μg·mL^−1^). These cultures were used to inoculate an expression culture in LB medium supplemented with ampicillin (100 μg·mL^−1^) to an initial OD_580 nm_ = 0.05. The cultures were grown at 37 °C until they reached logarithmic phase (OD_580 nm_ = 0.5–0.8) and gene expression was induced by addition of isopropyl‐β‐D‐thiogalactosid (IPTG) to a final concentration of 0.4 mm. After 2 h, cells were harvested by centrifugation at 4000 ***g*** and 4 °C for 20 min and stored at −20 °C.

### SDS/PAGE, zymography and immunodetection

Proteins were analysed by sodium dodecyl sulphate polyacrylamide gel electrophoresis (SDS/PAGE) under denaturation conditions on 12% (w/v) gels as described by Laemmli 28. Esterase activity in SDS/PAGE gels was detected by zymography using the fluorescent substrate 4‐methylumbelliferyl butyrate (MUB) 29. The proteins transferred from SDS/PAGE gel to the polyvinylidene difluoride membranes by western blotting 30 were detected using anti‐His(C‐term)‐HRP antibodies (Invitrogen) according the manufacturers' instructions. The protein concentration was determined by the method of Bradford with bovine serum albumin as a standard 31.

### Cellular localisation, purification and biochemical characterisation

#### Cellular localisation

Gene *pa2949* was subcloned from pET‐*pa2949* into pBBR1mcs‐3 behind the *lac* promoter using *Xba*I and *Sac*I restriction sites yielding plasmid pBBR‐*pa2949*. *E. coli* DH5α cells harbouring pBBR‐pa2949 were cultivated overnight in LB medium supplemented with tetracycline (10 μg·mL^−1^) at 37 °C. The cells were harvested by centrifugation (1 min, 19 000 ***g***, and 4 °C), resuspended in 100 mm Tris‐HCl buffer (pH 8), disrupted by sonication and total cell membranes were isolated by ultracentrifugation (30 min, 180 000 ***g***, 4 °C) 29, 32.

#### Purification

The total membrane fraction containing PA2949 with a C‐terminal His_6_‐tag was solubilised with 1% (w/v) Triton X‐100 by gentle agitation overnight at 4 °C. Solubilised membranes were subjected to ultracentrifugation (30 min, 180 000 ***g***, 4 °C) and PA2949 was purified from the supernatant by immobilised metal affinity chromatography (IMAC) using Ni‐NTA agarose (Qiagen) 33. All buffers used were supplemented with (1% w/v) Triton X‐100 to keep PA2949 in soluble form. Purified PA2949 samples eluted from the Ni‐NTA column were transferred into Tris‐HCl buffer (100 mm, pH 8) containing 1% (v/v) Triton X‐100 by gel filtration using PD‐10 column (GE Healthcare, Solingen, Germany) according to the manufacturers' protocol. The purified protein was then stored at room temperature.

#### Enzyme activity assays

Enzymatic activities were determined in 96‐well microplates by adding 5 μL of enzyme sample to 200 μL of substrate with *p*‐nitrophenyl hexanoate (*p*NPC_6_) for esterase and *p*‐nitrophenyl palmitate (*p*NPP) for lipase activity 1.

#### Temperature optimum

Esterase activities were measured over a range of temperatures from 10 °C to 70 °C as described previously 34. Assays were performed in a 96‐well microplate by adding 2 μL of enzyme sample to 200 μL of *p*NPC_6_ substrate.

#### pH and organic solvents stability

Esterase activities of PA2949 incubated for 1 h with buffers of a pH range from 3 to 10.5 34 or for 3 h with various organic solvents (dimethyl sulfoxide, *N*,* N*‐dimethyl formamide, methanol, acetonitrile, ethanol, acetone, propan‐2‐ol, diethyl ether, hexane, toluene) 34 were measured in a 96‐well microplate by adding 2 μL of enzyme sample to 200 μL of *p*NPC_6_ substrate.

#### Inhibition

The inhibition of PA2949 was tested according Asler *et al*. 35 using THL, PMSF, paraoxon (all dissolved in propan‐2‐ol) and the EDTA (dissolved in H_2_O). Inhibition of PA2949 was performed by incubating enzyme aliquots with the inhibitors for 3 h at the room temperature and subsequent determination of enzyme activity using *p*NPC6 as the substrate.

## Results and Discussion

### Open reading frame PA2949 of *P. aeruginosa* PA01 encodes a putative lipase

**Figure 1:**
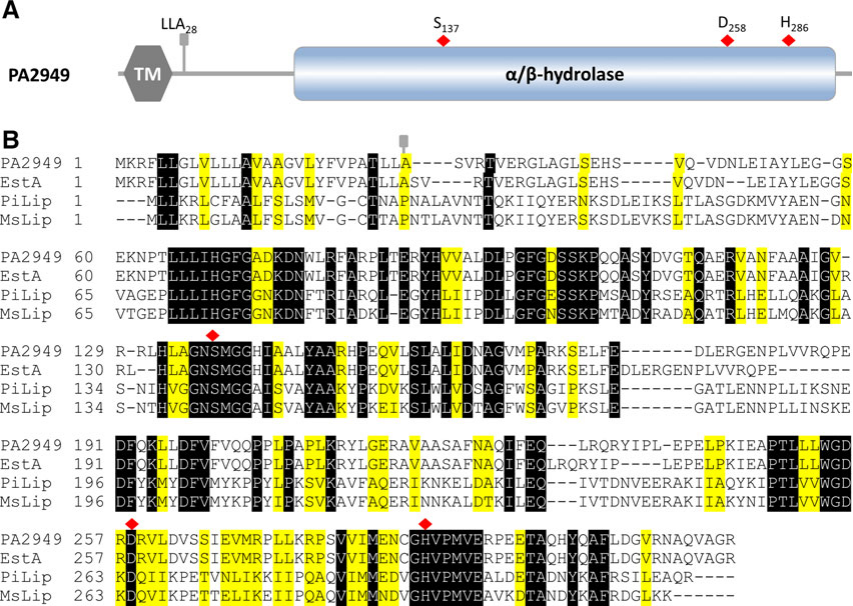
Sequence analysis of PA2949. (A) Molecular organisation of PA2949 from *P. aeruginosa* PA01 showing putative transmembrane domain (TM, amino acids 4‐24) and catalytic α/β‐hydrolase domain (amino acids 65–299). The cleavage site for the predicted signal peptide (LLA) and the putative active site residues (Ser, Asp, His) are marked by the grey pin and the red diamonds respectively. (B) Sequence alignment of PA2949 with EstA from *P. aeruginosa* 1001 19, 20, *Moraxella* sp. lipase MsLip 37 and *Psychrobacter immobilis* lipase PiLip 50. Residues identical and similar in at least three sequences were shaded in black and yellow respectively.

By searching the *Pseudomonas* genome database (www.pseudomonas.com) 36 we have identified about hundred genes of *P. aeruginosa* PA01 encoding putative lipolytic enzymes, among them ORF *pa2949*. This gene of 948 bp length encodes a protein of M_r_ 34.8 kDa with a predicted Abhydrolase_6 Pfam domain (PF12697) spanning residues 65–299 (Fig. 1A). A blast search revealed homology of PA2949 with known esterases and lipases, namely EstA from *P. aeruginosa* 1001 19, 20, and two lipases from psycrophilic bacteria, *Moraxella sp*. lipase 37 and *Psychrobacter immobilis* lipase 38 (Fig. 1B). The genes encoding *pa2949* from *P. aeruginosa* PA01 and *estA* from *P. aeruginosa* 1001 share 99% identity (data not shown), and the protein sequences are identical (Fig. 1). The amino acid sequences of *Moraxella* sp. and *P. immobilis* lipases are ~ 50% similar to the one of PA2949. The sequence alignment revealed the strictly conserved amino acid Ser137 embedded in the conserved GXSXG‐lipase motif, as well as Asp258 and His286 predicted to form the catalytic triad of PA2949.

### Expression of PA2949 in *E. coli* BL21(DE3)

**Figure 2:**
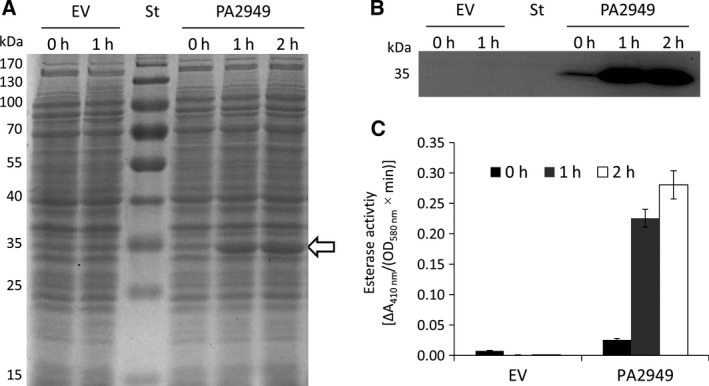
Expression and esterase activity of PA2949. (A) Coomassie Blue‐stained SDS gel (12%) after separation of extracts from *E. coli* BL21(DE3) cells carrying empty vector (EV, pET22b) or PA2949 expression vector (pET‐pa2949). The gel was loaded with equal amount of cell extracts collected before induction (0 h) and 1 or 2 h after induction with IPTG. Molecular weights of standard proteins (St) are indicated on the left; the black arrow indicates overexpressed PA2949. (B) Western blot of samples shown in A using anti‐His‐tag antibodies. (C) Esterase activity of cell extracts from samples shown in Fig. 2A. The results ± standard deviations are means of three independent experiments, each set in triplicate.

In order to obtain enzymatically active and soluble PA2949, we constructed a heterologous expression system using *E. coli* BL21(DE3) carrying plasmid pET‐*pa2949*. Bacteria were grown at 37 °C and expression of *pa2949* was induced by addition of 0.4 mm IPTG. SDS/PAGE and western blot analyses revealed expression of a protein with an estimated molecular weight of 35 kDa. In parallel, we measured significantly increased esterase activity in the cell lysate of the expression strain compared with the strain carrying the empty vector (Fig. 2). Additionally, esterase activity of the 35 kDa protein was detected by zymographic analysis. Interestingly, PA2949 did not show activity with palmitic acid *p*‐nitrophenyl ester considered as a typical lipase substrate. In conclusion, we could demonstrate that PA2949 of *P. aeruginosa* can be functionally expressed in *E. coli* BL21(DE3) and displays esterase but not lipase activity.

### PA2949 is localised in the membrane of *E. coli*

The subcellular localisation of a protein must be considered to develop an efficient purification protocol 39. As cellular localisation was unknown for PA2949 and its homologues, we first performed a bioinformatic analysis revealing either a putative N‐terminal type I signal peptide spanning amino acids 1–28 or a putative transmembrane helix spanning amino acids 4–24 suggesting a periplasmic, extracellular or membrane localisation. Many membrane proteins containing transmembrane helices display anomalous migration in SDS/PAGE 40 that is often heat inducible 41, and caused by differences in binding of SDS to heat‐treated and untreated forms of the protein. Hence, we have tested the effect of temperature on the electrophoretic mobility of PA2949 and have shown that PA2949 migrated faster after incubation at 99 °C prior to electrophoresis compared to the sample incubated at room temperature indicating that it is a membrane protein (data not shown).

**Figure 3:**
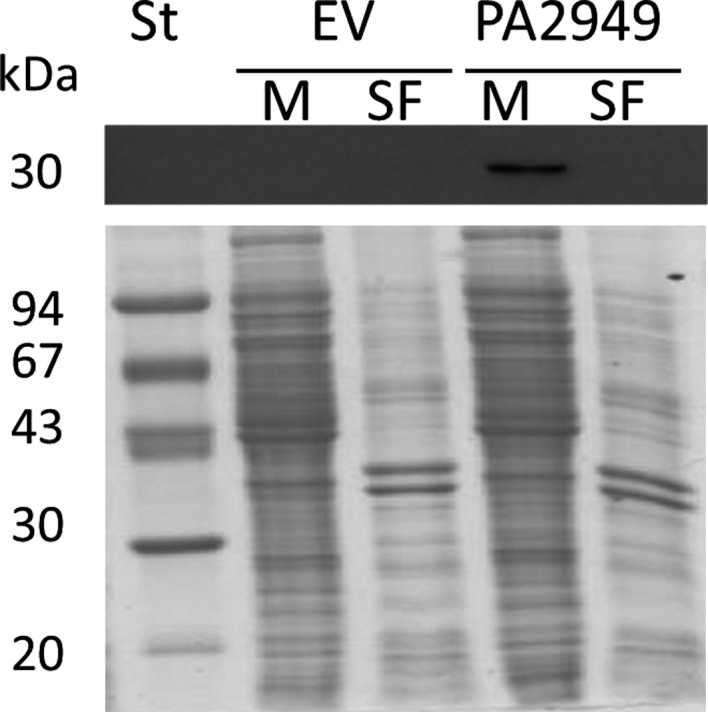
Subcellular localisation of recombinant PA2949 in *E. coli* DH5α. The membranes (M) and soluble fraction (SF) of *E. coli* DH5α carrying pBBR‐pa2949 or pBBR1mcs‐3 (empty vector, EV) were separated by ultracentrifugation and equal amount of proteins from each cell fraction were analysed by western blotting using anti‐His‐tag antibodies (upper panel) and by SDS/PAGE (lower panel). Molecular weights of protein standard (St) are indicated on the left.

Therefore, we have experimentally separated membranes and soluble fraction of *E. coli* cells expressing PA2949. For this experiment, we expressed PA2949 in *E. coli* DH5α under the control of the weak *lac* promoter rather than the strong T7 promoter to avoid overloading of membranes and prevent mislocalisation 42. The gene *pa2949* was subcloned from plasmid pET‐*pa2949* into the broad host range vector pBBR1mcs‐3 43 and expression was performed in *E. coli* DH5α without induction. Cells were disrupted by sonication, membranes were isolated by ultracentrifugation, analysed by SDS/PAGE and western blotting and PA2949 was detected solely in the membrane fraction (Fig. 3). Thus, recombinant PA2949 expressed in *E. coli* is localised in the cell membrane, however, the localisation of PA2949 in the homologous host *P. aeruginosa* is still unknown. As *E. coli* and *P. aeruginosa* belong to the same class of Gamma‐proteobacteria and share evolutionarily conserved signal recognition particles and Sec‐translocation systems for targeting membrane proteins 44 we predict membrane localisation of PA2949 in *P. aeruginosa* as well.

### Purification and biochemical characterisation of PA2949

**Figure 4:**
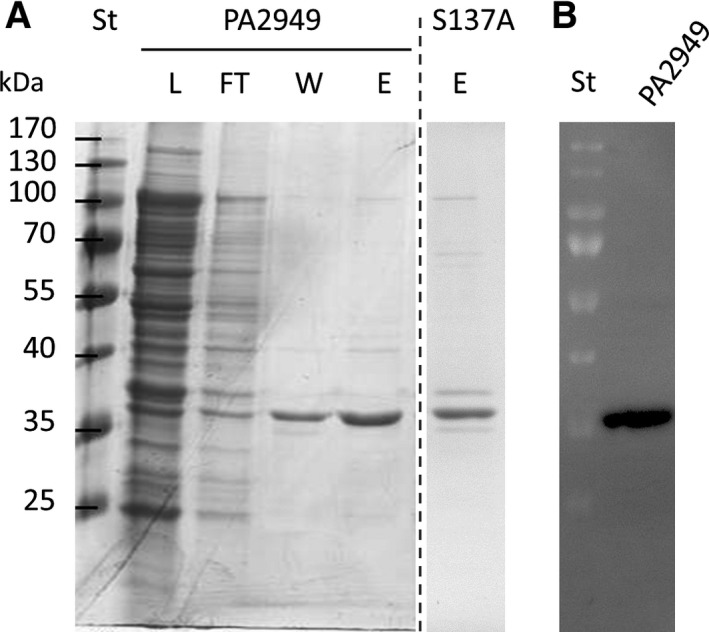
Purification of recombinant PA2949 from *E. coli* BL21(DE3). (A) Coomassie Blue‐stained SDS‐gel (12%) showing fractions obtained by purification of PA2949 and its catalytic inactive variant PA2949_S137A using a Ni‐NTA column. L, load fraction containing membranes of *E. coli* BL21(DE3) expressing PA2949 solubilised with Triton X‐100, FT, flow through; W, fraction after washing with buffer containing 60 mm imidazole and E, fraction eluted from the column with buffer containing 250 mm imidazole. Molecular weights of protein standard (St) are indicated on the left. The fraction containing PA2949_S137A is shown on a separate SDS gel (indicated by a dashed line). (B) Zymogram indicating esterase activity of purified PA2949 (elution fraction shown in A) monitored under UV light using the fluorescent substrate 4‐methylumbelliferyl butyrate.

Purification of membrane proteins requires the usage of detergents for solubilisation as well as to prevent subsequent protein aggregation by stabilising hydrophobic domains, which are naturally embedded in the membrane 45. Here, we have selected the nonionic detergent Triton X‐100 commonly used for purification of membrane proteins from *E. coli* 46 to extract PA2949 from the membranes. Initially, the total membrane fraction of *E. coli* BL21(DE3) expressing PA2949 was incubated for 1 h at room temperature with Triton X‐100 at concentrations exceeding the critical micellar concentration. Although, Triton X‐100 in a concentration range from 0.5 to 2% (w/v) did not reduce the esterase activity of PA2949, the protein was not quantitatively solubilised from the membranes (results not shown). Almost quantitative extraction of PA2949 was achieved after overnight incubation of membranes with detergent without losing esterase activity. Solubilisation of membranes with Triton X‐100 and subsequent purification using immobilised metal affinity chromatography yielded ~ 1 mg/*L*_culture_/OD_580 nm_ of pure PA2949 (Fig. 4). Purified PA2949 preparation had 198.8 ± 5.1 U·mg^−1^‐specific esterase activity measured with *p*‐nitrophenyl hexanoate at 30 °C that corresponds to approximately 60–70% of total esterase activity of membrane fraction of *E. coli* BL21(DE3)‐expressing PA2949. Incubation of PA2949 with various buffers (pH 3.0–10.5) revealed that activity was best retained in Tris‐HCl buffer pH 8.0 at room temperature (Table 2), whereas storage at 4 °C or freezing (also in the presence of 30% glycerol) lead to the precipitation and inactivation of PA2949.

**Table 2:** pH stability of PA2949

pH	Activity ± SD (%)
3.0	40.1 ± 2.0
4.5	60.2 ± 4.3
6.0	80.4 ± 3.7
7.5	88.7 ± 7.8
8.0	100.0 ± 6.7
8.5	87.4 ± 8.7
9.0	81.1 ± 6.7
9.5	62.5 ± 12.6
10.5	42.3 ± 9.1

Esterase activities ± standard deviations are means of three independent experiments, each set in triplicate.

**Figure 5:**
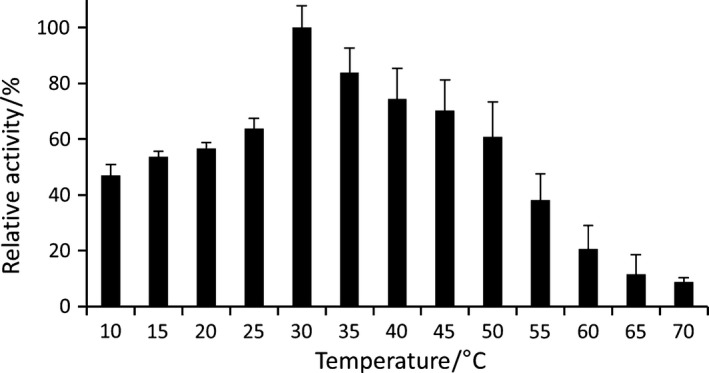
Optimum temperature of PA2949. Esterase activities of purified PA2949 measured at respective temperatures are shown as relative activities compared with the highest activity measured at 30 °C with 100% activity corresponding to the specific activity of the wild‐type (198.8 ± 5.1 U·mg^−1^). The results ± standard deviations are means of three independent experiments, each set in triplicate.

The similarity of the PA2949 amino acid sequence with psychrophilic esterases (Fig. 1) prompted us to test if PA2949 retained its activity also at low temperatures. Determination of esterase activities at temperatures ranging from 10 °C to 70 °C revealed 30 °C as the optimal temperature (Fig. 5). Interestingly, even at a temperature of 10 °C PA2949 retained 47% of its activity measured at 30 °C. This data indicate that PA2949 behaves similar to psychrophilic rather than mesophilic enzymes that are usually inactive at low temperatures 47.

Stability in the presence of organic solvents is an essential property of enzymes used as biocatalysts for organic synthesis 13. Membrane proteins usually contain membrane‐embedded domains and soluble domains protruding into the surrounding water milieu, which are differently resistant to hydrophobic (high log *P*) and hydrophilic (low log *P*) solvents 48. Therefore, we have tested the stability of PA2949 after incubation with various solvents (Table 3). Notably, PA2949 was fairly stable in most of the tested solvents, some of them, for example, diethyl ether and methanol even enhanced its activity, but some hydrophilic solvents (acetonitrile, ethanol and propan‐2‐ol) severely reduced esterase activity.

**Table 3:** Stability of PA2949 in the presence of various organic solvents

Organic solvent	Concentration (%, v/v)	Log *P*	Residual activity ± SD (%)^a^
DMSO	30	−1.300	82.2 ± 4.7
*N*,*N*‐Dimethyl formamide	30	−1.000	26.0 ± 2.7
Methanol	30	−0.760	111.0 ± 11.1
Acetonitrile	30	−0.330	0.0 ± 0.0
Ethanol	30	−0.240	5.4 ± 0.2
Acetone	30	−0.230	79.1 ± 5.6
Propan‐2‐ol	30	0.074	8.1 ± 0.4
Diethyl ether	30	0.850	125.6 ± 8.8
Hexane	5	3.500	106.9 ± 5.8
Toluene	5	2.500	65.1 ± 2.7

a Residual esterase activities are expressed as a percentage of PA2949 activity in buffer without organic solvent. ± standard deviations are means of three independent experiments, each set in triplicate.

**Figure 6:**
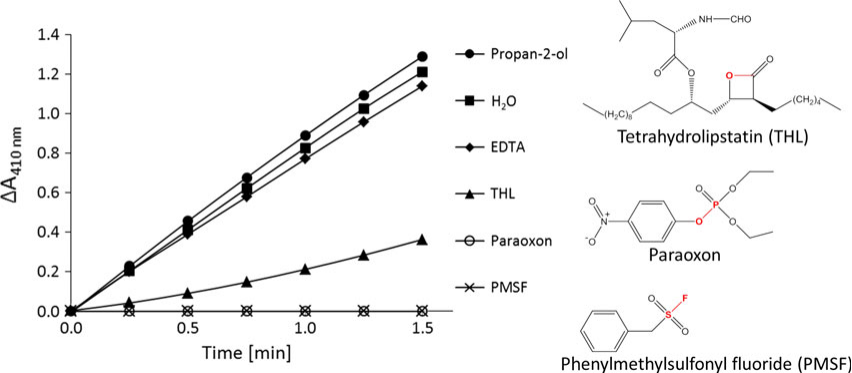
Inhibition of PA2949. Residual esterase activities were determined after preincubation for 3 h of 1.7 μm of PA2949 with 10 mm EDTA, 2 mm THL, 1 mm paraoxon and 1 mm PMSF at room temperature. Inhibited PA2949 samples (15 μg) were incubated with 100 μL of substrate at 30 °C and residual esterase activity was measured. PA2949 samples treated with propan‐2‐ol and water represent noninhibited controls. On the right, the chemical structures of inhibitors THL, paraoxon and PMSF are shown with the bonds to be hydrolysed indicated in red. The results ± standard deviations are means of three independent experiments, each set in triplicate. The standard deviations were below 8%.

Binding of substrate‐mimicking inhibitors to the enzyme active site can provide valuable data for understanding the catalytic mechanism 49 as well as regio‐ and enantio preference 50 of lipases. We thus determined the inhibition kinetics of PA2949 using typical lipase inhibitors containing long hydrophobic acyl chains, namely tetrahydrolipstatine (THL) 51, the short acyl chain arylesterase inhibitors paraoxon 52, phenylmethylsulfonyl fluoride (PMSF) 53 and an inhibitor of metal‐dependent enzymes, ethylenediaminetetraacetic acid (EDTA) 54. Resistance towards EDTA indicated that PA2949 is not a metalloenzyme (Fig. 6). All three inhibitors targeting the catalytic serine residue (THL, PMSF and paraoxon) inhibited PA2949 activity, although to a different degree (Fig. 6). Incubation of PA2949 with THL for 3 h resulted in 28% of residual activity, whereas THL, PMSF and paraoxon completely abolished PA2949 activity indicating irreversible inhibition. Phosphonate or sulphonate inhibitors covalently linked to the catalytic serine, mimic the first tetrahedral intermediate (before dissociation of alcohol moiety) and the second tetrahedral intermediate (after dissociation of alcohol moiety) formed during ester hydrolysis respectively 10. These results provided further evidence that PA2949 contains in its active site a nucleophlic serine. Using site‐directed mutagenesis, we constructed variant PA2949 S137A, which was purified (Fig. 4), but did not show any esterase activity. These results are in agreement with the bioinformatic prediction of Ser137 as the catalytic residue. Additionally, our data suggest a rather narrow active site of PA2949 because the bulky inhibitor molecule THL apparently could not efficiently bind to the active site. The preparation of stable PA2949‐PMSF and PA2949‐paraoxon complexes we have described here will be used for further crystallographic and kinetic studies.

### Biotechnological potential of PA2949

PA2949 from *P. aeruginosa* PA01 is homologous to the esterase EstA from *P. aeruginosa* 1001, which was previously shown to hydrolyse racemic β‐acetylthioisobutyrate methyl ester to form enantiopure DAT 19, 20, an important intermediate in the synthesis of pharmaceuticals 16. However, heterologous expression of enzymatically active EstA turned out to be difficult because of poor solubility and low production yield 20. EstA was predicted to be a soluble protein with an N‐terminal signal sequence instead of a transmembrane helix 19, 20. This misleading assumption resulted in the construction of an expression system where maltose‐binding protein (which was shown to enhance the solubility of proteins) was fused to the N‐terminus of EstA resulting in blocking of EstA secretion and expression of only poorly active enzyme 20. Using the *pa2949* gene encoding the full‐length protein with C‐terminal His6‐tag we have successfully expressed mg/L_culture_ quantities of active PA2949 located in the membrane of the common laboratory strain *E. coli*. Subsequent extraction with the nonionic detergent Triton X‐100 allowed for the efficient purification of up to 70% of PA2949 from membranes and enabled storage at room temperature without loss of activity. The biochemical characterisation of PA2949 revealed 30 °C as the optimal temperature for catalysis (Fig. 5), high stability at pH 7.5–8.5 (Table 2), and notable resistance to various organic solvents (Table 3). Interestingly, a study of DAT production with whole cells of *P. putida* MR‐2068, which expressed a PA2949 homologue revealed pH 7.5 and 45 °C as the best conditions 55. At higher temperatures and in alkaline pH spontaneous hydrolysis of the racemic β‐acetylthioisobutyrate methyl ester was observed, thereby reducing the optical purity of DAT 55. In summary, PA2949 possesses biochemical properties, which match the requirements for the enzymatic synthesis of DAT as well as its subsequent extraction with organic solvent.

## Author contributions

FK, KEJ, RBS and JG conceived and supervised the study; FK, SW and KEJ designed experiments; FK, FB and MC performed experiments; FK, RBS and JG analysed data; FK, KEJ and RBS wrote the manuscript.
